# Supplementary material for: Progress toward Polymerization Reaction Monitoring with Different Dienes: How Small Amounts of Dienes Affect ansa-Zirconocenes/Borate/Triisobutylaluminium Catalyst Systems
Source: Polymers (Basel). 2022 Aug 9;14(16):3239. doi: 10.3390/polym14163239 (PMC9414859; doi:10.3390/polym14163239)
Supplement: Supplementary file 1 [file polymers-14-03239-s001.zip › polymers-1860849-supplementary.pdf]

Supporting information

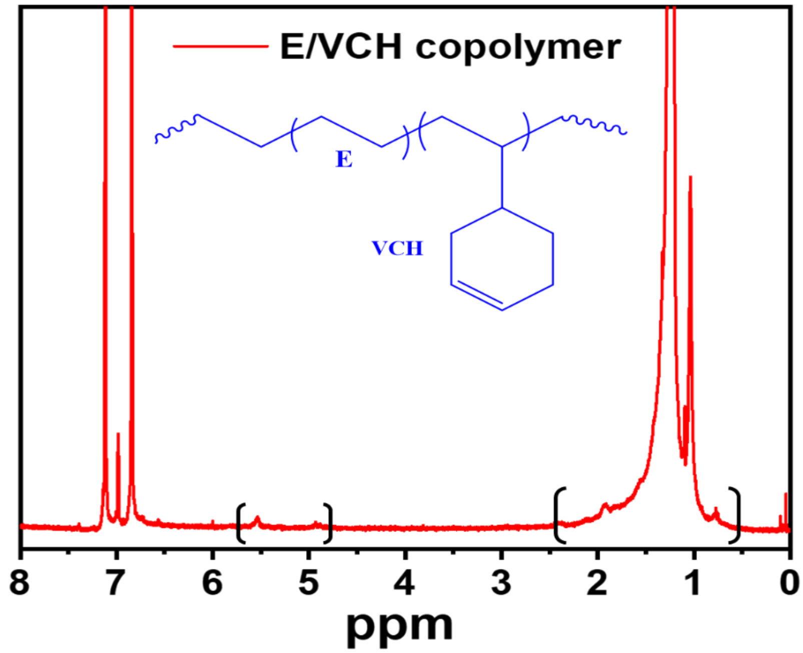

Figure S1a.

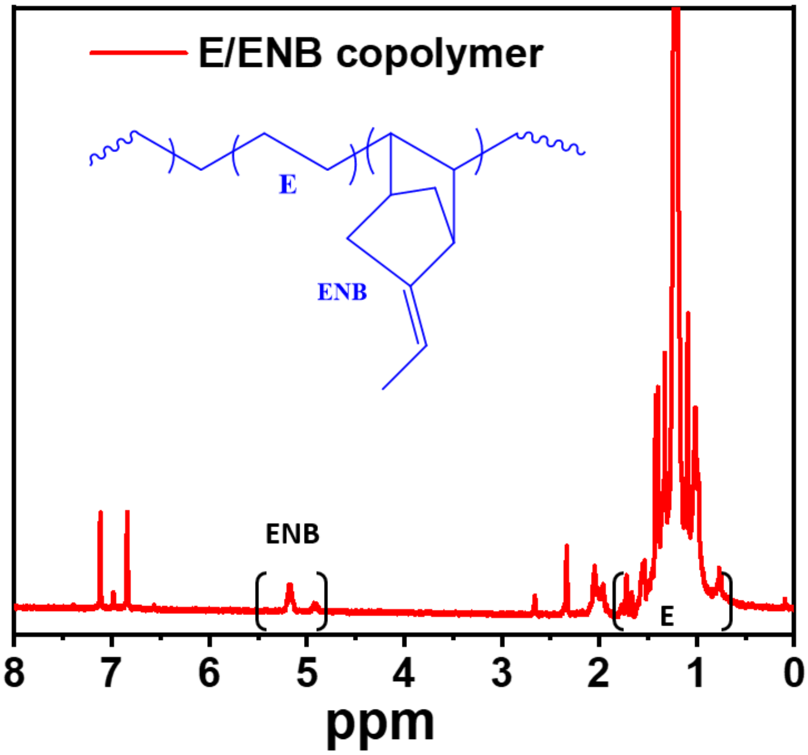

Figure S1b.

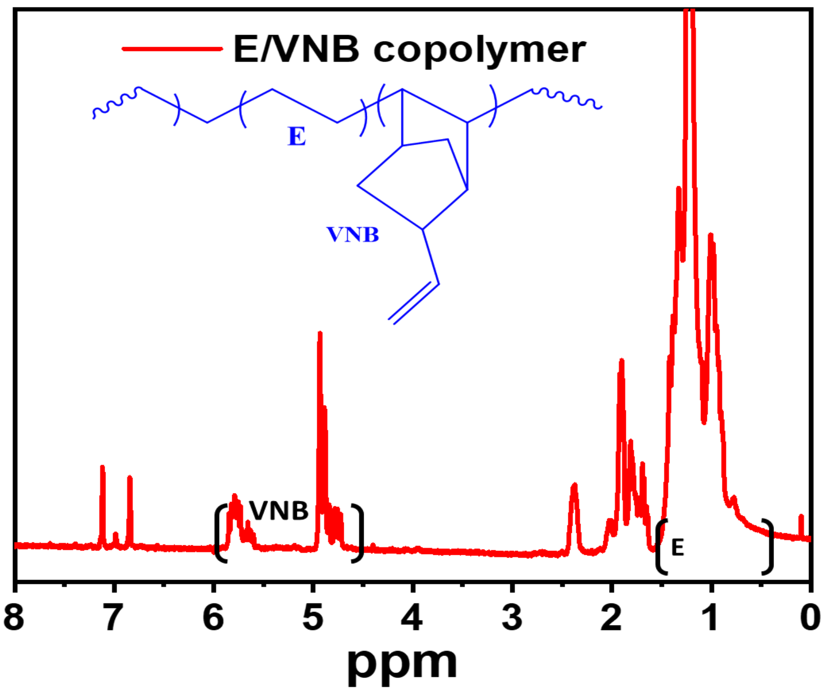

Figure S1c.

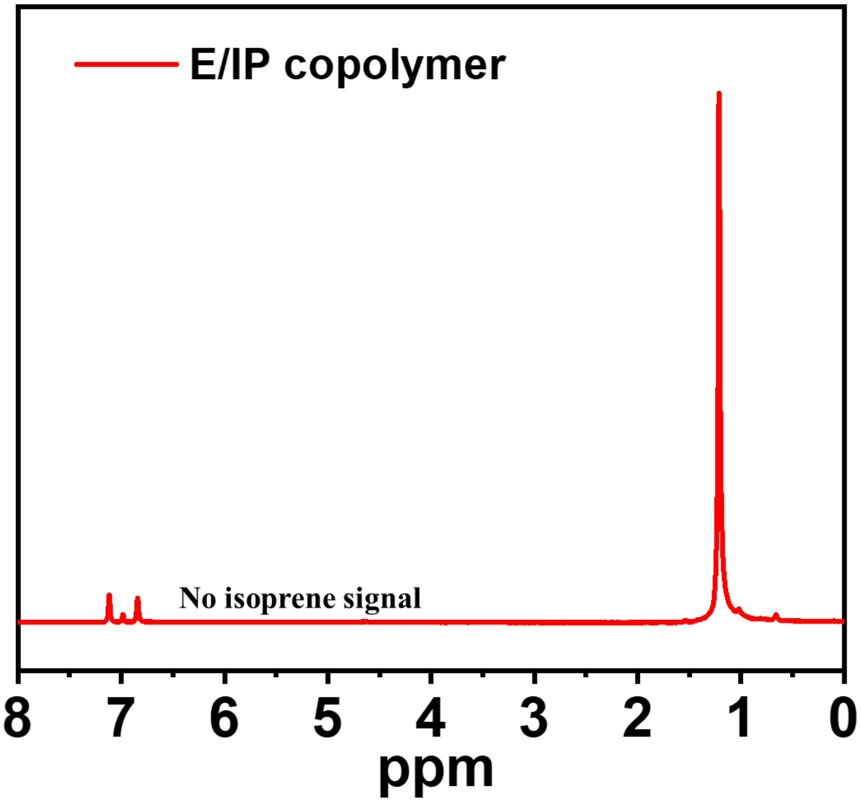

Figure S1d.

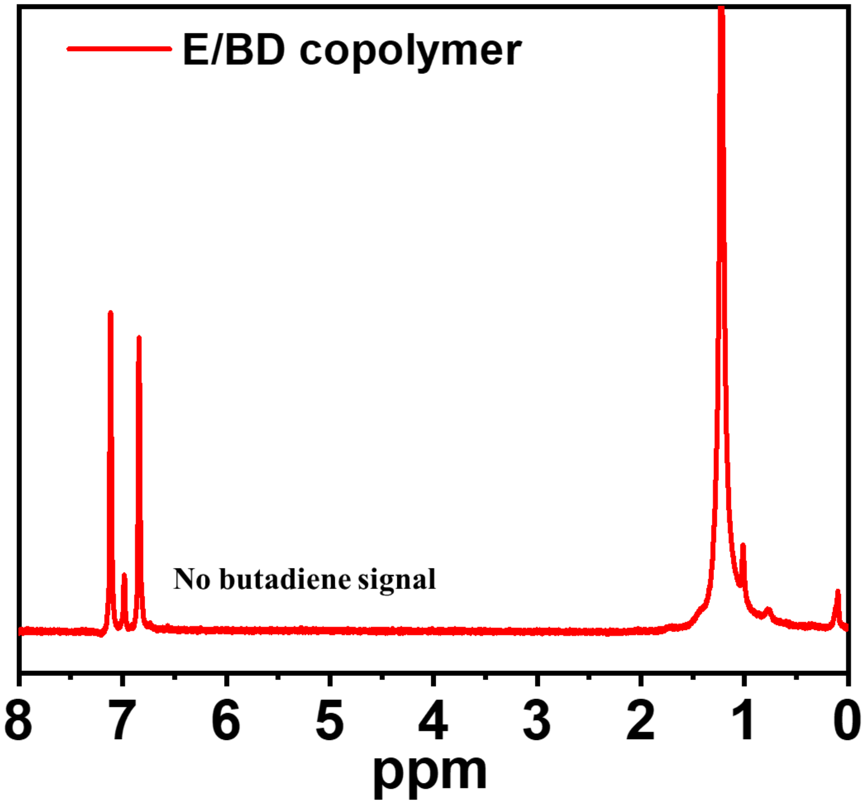

Figure S1e.

Figures S1a to S1e, the structure of E/diene copolymers

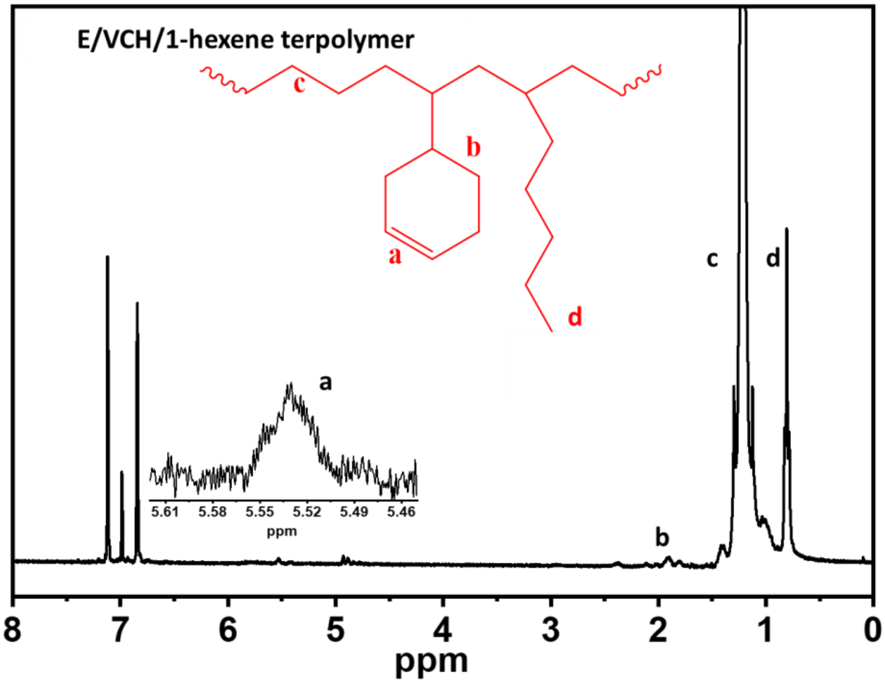

Figure S2a.

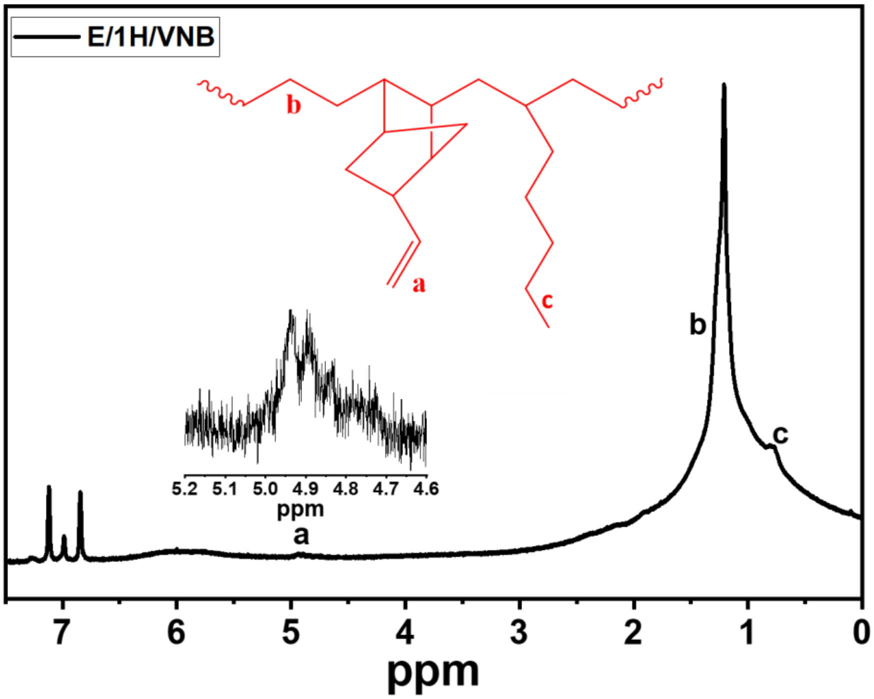

Figure S2b.

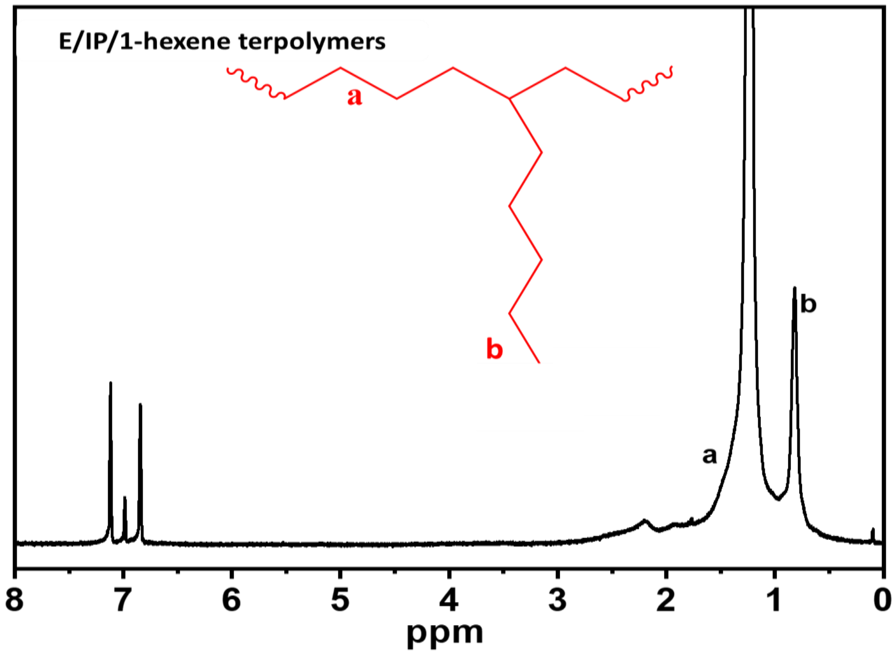

Figure S2c.

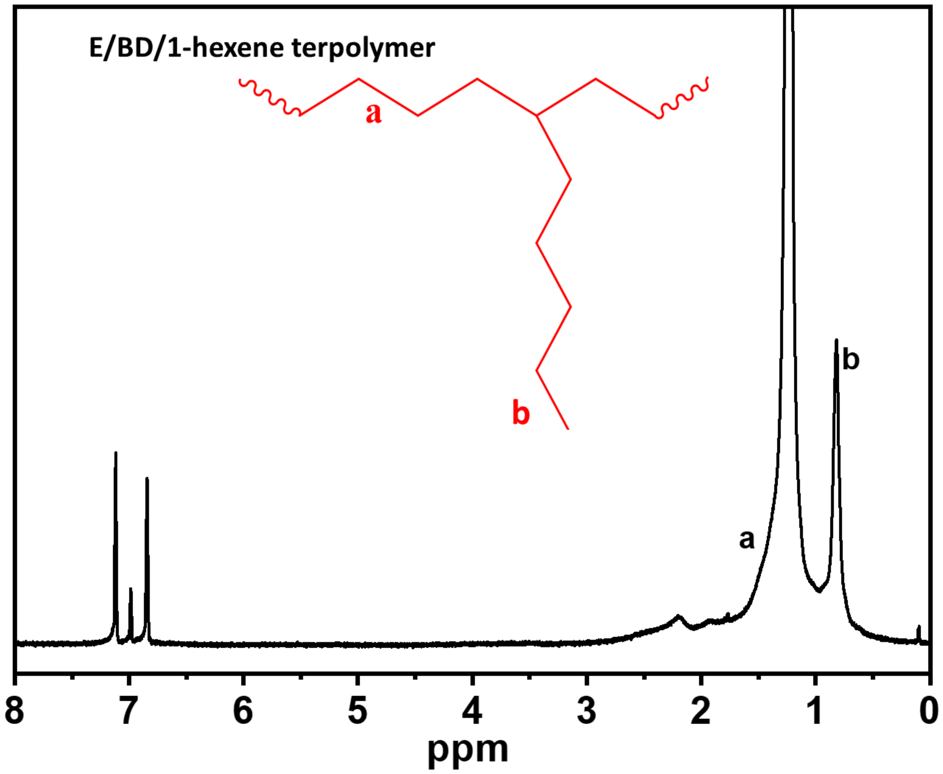

Figures S2d

Figures S2a to S2d, the structure of E/diene/1-hexene terpolymers

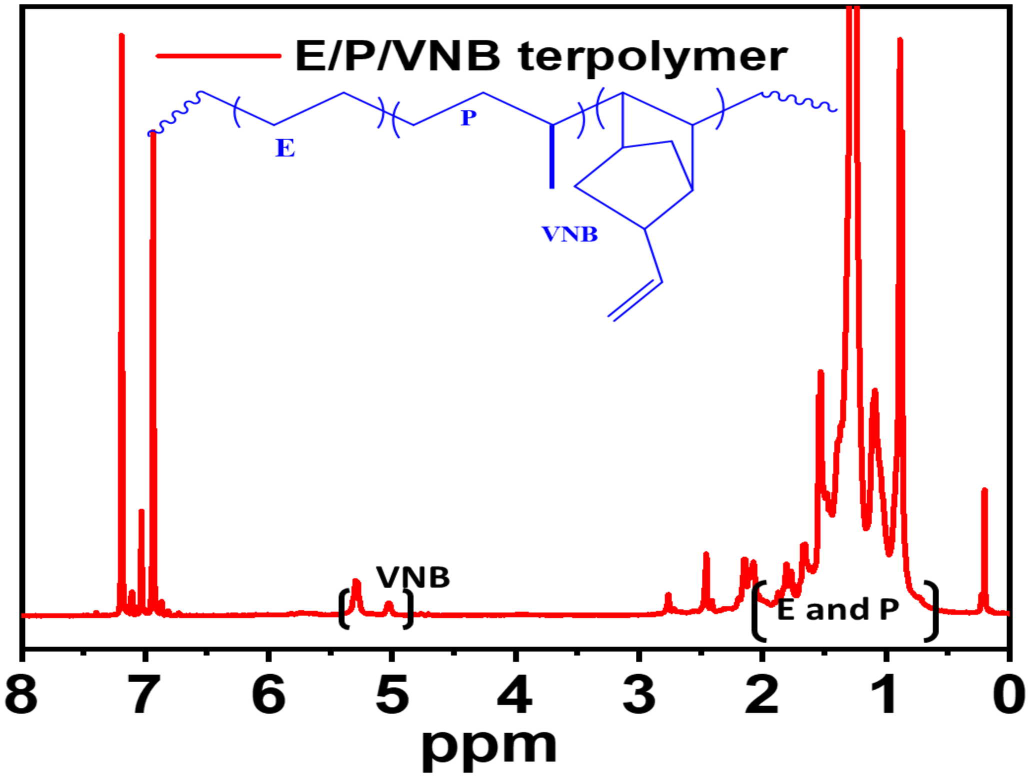

Figure S3a.

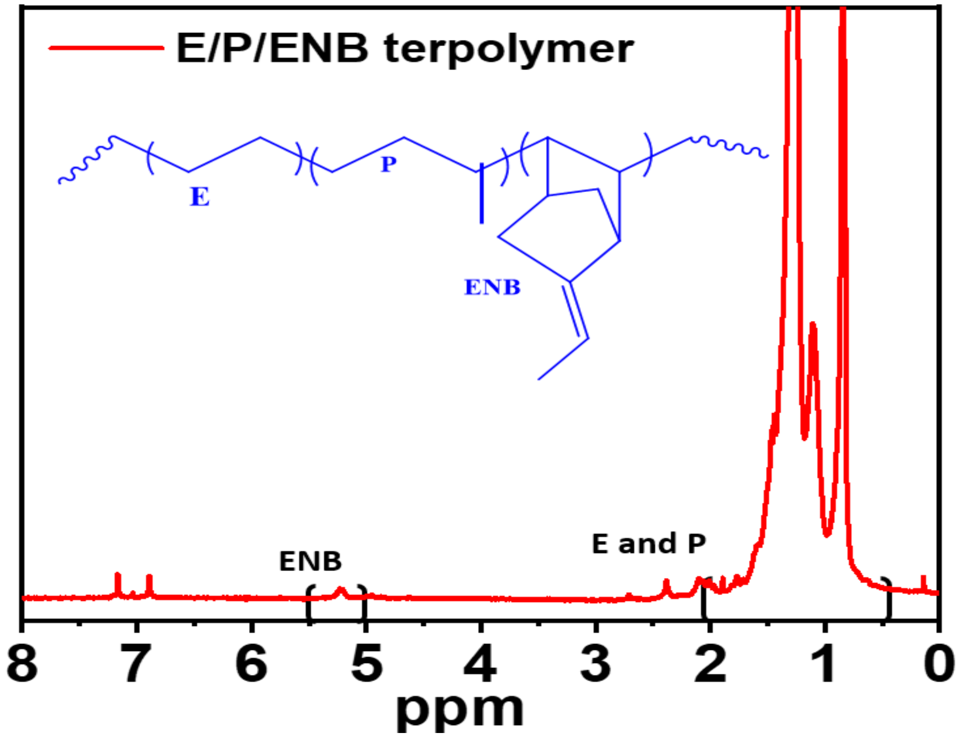

Figure S3b.

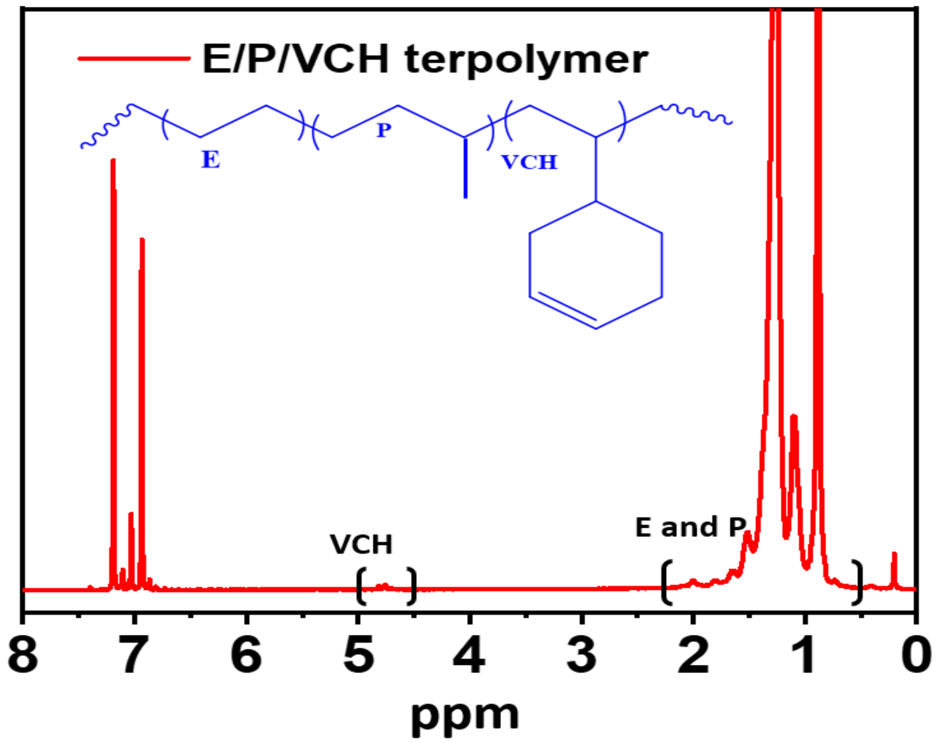

Figure S3c.

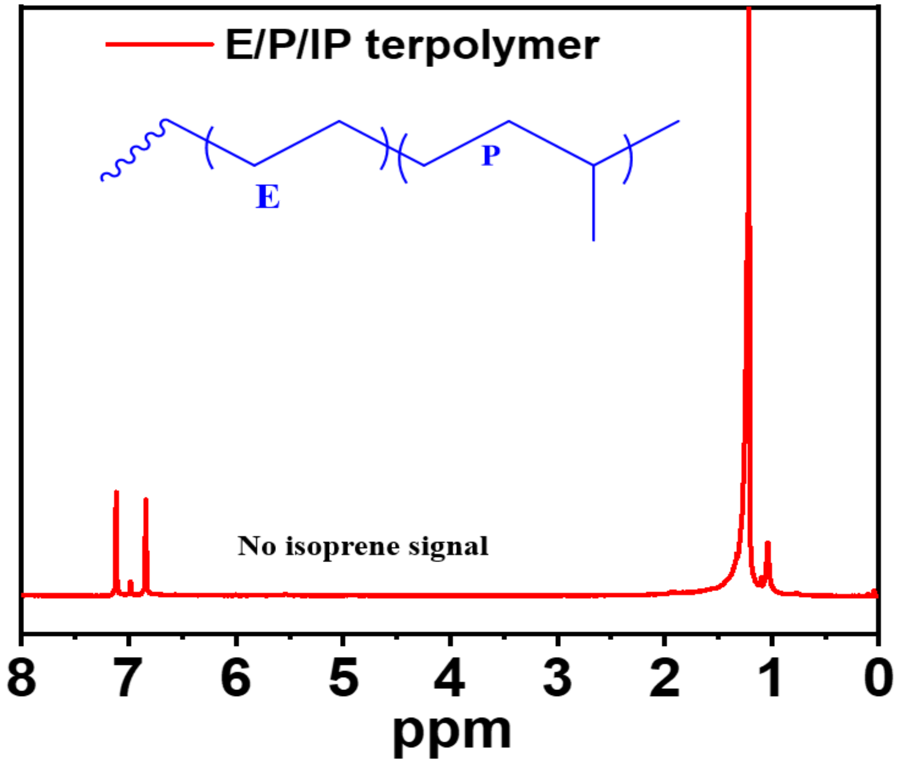

Figure S3d.

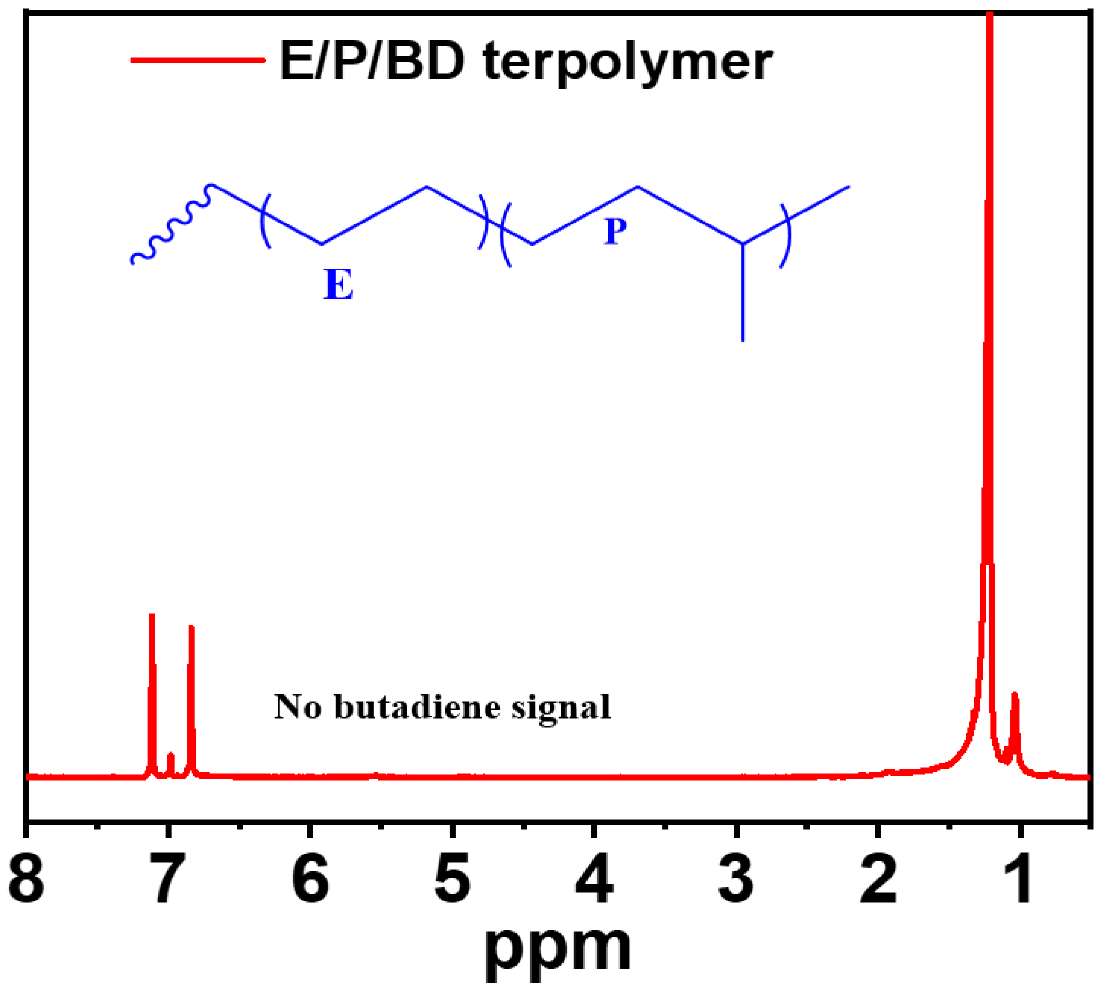

Figure S3e.

Figures S3a to S3e, the structure of E/diene/propylene terpolymers
